# Prospecting *Russula senecis*: a delicacy among the tribes of West Bengal

**DOI:** 10.7717/peerj.810

**Published:** 2015-03-10

**Authors:** Somanjana Khatua, Arun Kumar Dutta, Krishnendu Acharya

**Affiliations:** Molecular and Applied Mycology and Plant Pathology Laboratory, Department of Botany, University of Calcutta, Kolkata, West Bengal, India

**Keywords:** Antimicrobial property, Antioxidant activity, Edible mushroom, HPLC, Internal Transcribed Spacer sequence, Molecular phylogeny, Taxonomy

## Abstract

*Russula senecis*, a worldwide distributed mushroom, is exclusively popular among the tribal communities of West Bengal for food purposes. The present study focuses on its reliable taxonomic identification through macro- and micro-morphological features, DNA barcoding, confirmation of its systematic placement by phylogenetic analyses, myco-chemicals and functional activities. For the first time, the complete Internal Transcribed Spacer region of *R*. *senecis* has been sequenced and its taxonomic position within subsection *Foetentinae* under series *Ingratae* of the subgen. *Ingratula* is confirmed through phylogenetic analysis. For exploration of its medicinal properties, dried basidiocarps were subjected for preparation of a heat stable phenol rich extract (RusePre) using water and ethanol as solvent system. The antioxidant activity was evaluated through hydroxyl radical scavenging (EC_50_ 5 µg/ml), chelating ability of ferrous ion (EC_50_ 0.158 mg/ml), DPPH radical scavenging (EC_50_ 1.34 mg/ml), reducing power (EC_50_ 2.495 mg/ml) and total antioxidant activity methods (13.44 µg ascorbic acid equivalent/mg of extract). RusePre exhibited antimicrobial potentiality against *Listeria monocytogenes*, *Bacillus subtilis*, *Pseudomonas aeruginosa* and *Staphylococcus aureus*. Furthermore, different parameters were tested to investigate its chemical composition, which revealed the presence of appreciable quantity of phenolic compounds, along with carotenoids and ascorbic acid. HPLC-UV fingerprint indicated the probable existence of at least 13 phenolics, of which 10 were identified (pyrogallol > kaempferol > quercetin > chlorogenic acid > ferulic acid, cinnamic acid > vanillic acid > salicylic acid > *p*-coumaric acid > gallic acid). Result from the present work suggests that the fraction, RusePre, may open novel prospect as a functional ingredient in antioxidant supplements and in drugs to treat infectious disease.

## Introduction

A recent estimation implies the existence of around 3 million fungi (Hawksworth, 2012) of which approximately 140,000 species pass the criteria as set by Chang & Miles (1992) to be considered as ‘Mushroom’ (Rajaratnam & Thiagarajan, 2012). Standing into the era of 21st century, our present knowledge on the described mushroom species by far accounts to be only 10% of total estimated mushroom diversity (Chang & Miles, 2004). Out of these 14,000 identified macrofungal species, about 650 have been recognized to possess medicinal properties (Thatoi & Singdevsachan, 2014). Thus, there is a recent trend among mycologists to document therapeutic value of mushrooms all around the globe and the present study is not an exception of that.

To meet the aim, West Bengal (21°38′-27°10′N latitude and 85°50′-89°50′E longitude) has been selected as study area due to its unique phyto-geographical feature. It is the only state in India which shares its topographical extension from Himalayas in the northern side to the Bay of Bengal in the southern with regions such as plateau and Ganges delta prevailing in between. These wide ranges of topographical feature, types of soils and substrata make the state to be ideal for hosting and flourishing rich diversity of mushrooms (Dutta & Acharya, 2014).

In the last 10 years, our research time has conducted extensive field work and inventoried a large number of wild mushrooms from different corners of the state with the active help from ethnic and tribal mushroom hunters of the regions (Pradhan et al., 2012; Dutta et al., 2013). Morphological and molecular investigation revealed that many of them are new to science (Acharya, Dutta & Pradhan, 2012; Dutta et al., 2014), new to the record for India (Dutta et al., 2011; Dutta et al., 2012a) and additionly to the macrofungal flora of West Bengal (Dutta et al., 2012b; Acharya et al., 2014), while some of the remaining mushrooms were revealed not to be currently documented as edible mushroom. In this context, an undocumented mushroom from our collection was taxonomically investigated, its systematic position was supported by the phylogenetic analysis, and its medicinal prospect was evaluated.

## Materials & Methods

### Mushroom sampling

During the field survey (2008–2012), several edible mushrooms were collected from the forest floor of West Bengal, India accompanied by tribal mushroom hunters of the regions. Among the basket of mushrooms which they usually gather for their regular dishes, a unique mushroom, commonly called “JHAL PATRA” (JHAL = because of its acrid taste; PATRA = Mushroom) was chosen and brought to the laboratory for thorough taxonomic investigation. Detailed microscopic work was performed using the protocol of Buyck & Adamcik (2011) and it was identified as *Russula senecis* S. Imai using standard literatures (Imai, 1938; Zhishu, Guoyang & Taihui, 1993; Das, 2009). Colour codes and terms (mostly) follow the Royal Botanic Gardens Edinburgh colour chart (Henderson, Orton & Watling, 1969). Scanning Electron Microscope (SEM) illustrations of basidiospores were carried out with a Zeiss EVO-MA10 electron microscope (Zeiss, Oberkochen, Germany) at the Centre for Research in Nanoscience and Nanotechnology, University of Calcutta, Kolkata, India. After thorough microscopic work, the specimen voucher had been deposited in Calcutta University Herbarium (CUH).

### Phylogenetic protocols

#### DNA extraction, polymerase chain reaction and sequencing

Genomic DNA was extracted from dried herbarium specimens (10–50 mg) using the ‘Fungal gDNA Mini Kit’ (Xcelris Genomics, Ahmedabad, India). ITS region 1 and 2, and the 5.8S rDNA, were amplified using universal primers pair ITS1 (5′ TCC GTA GGT GAA CCT GCG G 3′) and ITS4 (5′ TCC TCC GCT TAT TGA TAT GC 3′) (White et al., 1990). The DNA fragments were amplified on an Applied Biosystems^®^ 2,720 automated thermal cycler (Applied Biosystems, Carlsbad, California, USA) following the protocol as described by Abd-Elsalam et al. (2003) with little modifications. A hot start of 4 min at 94 °C was followed by 35 cycles consisting of 1 min at 94 °C, 1 min at 56 °C, 1 min at 72 °C, and a final elongation step of 7 min at 72 °C. PCR products were checked on 2% agarose gel stained with ethidium bromide. PCR products were purified using QIAquick^®^ Gel Extraction Kit (QIAGEN, Hilden, Germany) and was subjected to automated DNA sequencing based on Sanger dideoxy sequencing technique, on ABI3730xl DNA Analyzer (Applied Biosystems, Carlsbad, California, USA) using primers identical with amplification for ITS rDNA region. The newly generated sequences were then deposited in GenBank (www.ncbi.nlm.nih.gov) with the accession numbers KJ768982 and KP142981.

#### Taxon sampling

Twenty eight Internal Transcribed Spacer (ITS) nrDNA sequences representing nineteen species were used in the analyses, of which two sequences of *Russula senecis* S. Imai were generated as part of this study. The sequences represent sixteen species of *Russula* distributed over five subgenus, namely *Compacta* (Fr.) Bon (*Russula delica* Fr.), *Heterophyllidia* Romagn. (*Russula cyanoxantha* (Schaeff.) Fr. and *Russula virescens* (Schaeff.) Fr.), *Amoenula* Sarnari (*Russula amoenicolor* Romagn.), *Ingratula* Romagn. (*Russula* cf. *laurocerasi*, *Russula* cf. *subfoetens*, *Russula fellea* (Fr.) Fr., *Russula foetens* Pers., *Russula insignis* Quél., *Russula grata* Britzelm. (in the present study represented as *Russula laurocerasi* Melzer), *Russula ochroleuca* Fr., *Russula pulverulenta* Peck and *Russula senecis* S. Imai), *Russula* emend. Sarnari (*Russula emetica* (Schaeff.) Pers.) and *Incrustatula* Romagn. emend. (*Russula rosea* Pers.). *Stereum hirsutum* (Willd.) Pers., *Amylostereum laevigatum* (Fr.) Boidin, and *Bondarzewia mesenterica* (Schaeff.) Kreisel (here represented as *Bondarzewia Montana* (Quél.) Singer) were selected as outgroup taxa for rooting purpose following Buyck et al. (2008). The accession numbers of newly generated two ITS sequences of *R*. *senecis* and those pulled from GenBank for the purpose of conducting phylogenetic analysis for this study are cited in Fig. 3.

#### Phylogenetic analysis

Sequences were edited with the CodonCode Aligner software (CodonCode Corporation, Dedham, Massachusetts, USA). The newly generated two ITS1-5.8S-ITS2 sequences of *R*. *senecis* and those retrieved from GenBank were aligned with the help of ClustalX (Thompson et al., 1997) using the default setting. A final set of 28 sequences were aligned. The appropriate substitution model was determined using Bayesian information criterion (BIC) in MEGA6 (Tamura et al., 2013). The K2 + G model (with lowest BIC scores of 4931.469) was selected as the best-fit model.

Phylogenetic analyses was performed in MEGA6 (Tamura et al., 2013) using the Maximum Likelihood (ML) method based on the Kimura 2-parameter model. Initial tree(s) for the heuristic search were obtained by applying the Neighbor-Joining method to a matrix of pairwise distances estimated using the Maximum Composite Likelihood (MCL) approach. A discrete Gamma distribution was used to model evolutionary rate differences among sites (5 categories (+G, parameter = 0.5337)).

Beside ML method, phylogenetic analyses were also carried out using Neighbor-Joining (NJ) method (Saitou & Nei, 1987) to determine whether different methods (Maximum Likelihood versus Neighbor-Joining) alter the resulting phylogenetic tree. The evolutionary distances were computed using the Kimura 2-parameter method (Kimura, 1980) and are in the units of the number of base substitutions per site. The sum of branch length of the optimal tree was 1.12069257. In both the cases, all positions containing gaps and missing data were eliminated and a bootstrap test of 1,000 replicates was performed to obtain the percentage of replicate trees for clustering the associated taxa.

### Preparation of extract

Polyphenol rich fraction was extracted according to the method of Dasgupta et al. (2014). Dried and powdered basidiocarps of *R. senecis* were steeped with ethanol at 25 °C for 2 days to eliminate the alcohol soluble constituents such as coloured material, small organic molecules (steroid, terpenoids etc.) and fat. After filtration, the residue was then re-extracted with ethanol, as described above. The filtrate was air dried and extracted by stirring with distilled water at 100 °C for 7 h. Solvent was separated and 4 volume of ethanol was added slowly and kept at 4 °C overnight. Precipitate was discarded by centrifugation and supernatant was reduced in volume using a rotary evaporator (Butchi, Switzerland). This concentrated polyphenol rich extract of *R. senecis* (RusePre) was stored at −20 °C until further analysis.

### Antioxidant activity

Total antioxidant capacity assay was carried out as described by Prieto, Pineda & Aguilar (1999) with little modification (Mitra et al., 2014). The activity was expressed as the number of equivalents of ascorbic acid. The method described by Halliwell, Gutteridge & Arumo (1987) was followed for determination of hydroxyl radical scavenging activity. The radicals were generated by Fenton’s reaction in the presence of variable concentrations (1–10 µg/ml) of RusePre, and BHA was used as a positive control. The radical scavenging activity of RusePre (0.5–1.5 mg/ml) was evaluated using DPPH radicals based on the method by Shimada et al. (1992) where ascorbic acid was treated as standard. The ability of investigated extract to chelate ferrous ion was determined as described by Dinis, Mudaira & Alnicida (1994). Different concentrations of RusePre (0.05–0.2 mg/ml) were compared with EDTA, a positive control. A modified method of reducing power described by Oyaizu (1986) was considered. Various concentrations of RusePre (1–3 mg/ml) were mixed in a 1.5 mL reaction mixture, and the absorbance was measured at 700 nm. Ascorbic acid was used for comparison. The sample concentrations providing 50% of antioxidant activity or 0.5 of absorbance were calculated from the graphs of antioxidant activity percentages and regarded as EC_50_ value.

### Antimicrobial activity

#### Test bacteria

*Listeria monocytogenes* MTCC Code 657, *Salmonella typhimurium* MTCC Code 98, *Bacillus subtilis* MTCC Code 736, *Escherichia coli* MTCC Code 68, *Pseudomonas aeruginosa* MTCC Code 8158 and *Staphylococcus aureus* MTCC Code 96 were obtained from the culture collection of the Microbial Type Culture Collection and Gene Bank (MTCC), Institute of Microbial Technology, Chandigarh, India. They were incubated for 24 h by inoculation into nutrient broth.

#### Disk diffusion method

The determination of the inhibitory effect of RusePre on test bacteria was carried out by the agar-disc diffusion method (Bauer et al., 1966). Nutrient agar was poured into each sterilized petri dish (90 mm diameter) after injecting cultures (100 µl) of bacteria and medium was distributed homogeneously. Paper discs (5 mm) were loaded with 20 µl of 20 mg/ml concentrated RusePre. The impregnated discs were air dried before being place in on the petri dishes with the test microorganisms. Plates were incubated as per the bacterial requirements. Studies were performed in triplicate and the inhibition zones were compared with those of blank discs.

### Chemical composition

#### Mycochemical analyses

The content of total phenolic compounds in RusePre was estimated using Folin-ciocalteu reagent and gallic acid as standard (Singleton & Rossi, 1965). The results were expressed as µg of gallic acid equivalents per mg of dry extract. Total flavonoid content was determined using aluminium nitrate and potassium acetate. Quercetin (5–20 µg/ml) was used to calculate the standard curve (Park et al., 1997). The results were expressed as µg of quercetin equivalents per mg of dry extract. *β*-carotene and lycopene were estimated by measuring absorbance at 453, 505 and 663 nm (Nagata & Yamashita, 1992). Ascorbic acid was determined by titration against 2, 6-dichlorophenol indophenol dye (Rekha et al., 2012).

#### Determination of phenolic profile by HPLC

For quantitative analysis of phenolic compounds, a 3-level calibration curve was obtained by injection of known concentrations (10–50 µg/ml) of eleven standard compounds: gallic acid (*y* = 34.773*x* − 9.2238; *R*^2^ = 0.9991), chlorogenic acid (*y* = 13.776*x*–2.9025; *R*^2^ = 0.9993), vanillic acid (*y* = 19.225*x* + 0.2588; *R*^2^ = 0.9994), *p*-coumaric acid (*y* = 49.773*x* − 10.541; *R*^2^ = 0.9994), ferulic acid (*y* = 30.425*x* − 2.8188; *R*^2^ = 0.9995), myricetin (*y* = 5.0676*x* − 6.0375; *R*^2^ = 0.9937), salicylic acid (*y* = 4.4974*x* − 0.4763; *R*^2^ = 0.9994), quercetin (*y* = 5.2478*x* − 5.9763; *R*^2^ = 0.9954), cinnamic acid (*y* = 108.07*x* − 111.55; *R*^2^ = 0.9979), pyrogallol (*y* = 10.8*x* + 0.3333; *R*^2^ = 0.9999) and kaempferol (*y* = 18.667*x* − 80.875; *R*^2^ = 0.9997). The results were expressed as µg/mg of dry extract.

A 0.5 mg amount of RusePre was dissolved in 1 ml of methanol and water (1:1 v/v) and filtered through 0.2 µm filter paper. 20 µl filtrate was loaded on the HPLC system (Agilent, Santa Clara, California, USA). Separation was achieved on an Agilent Eclipse Plus C18 column (100 mm × 4.6 mm, 3.5 µm) using a flow rate of 0.8 ml/min at 25 °C. The mobile phase consisted of eluent A (acetonitrile) and eluent B (aqueous phosphoric acid solution, 0.1% v/v). A gradient program was used for elution: 0–2 min, 5% A; 2–5 min, 15% A; 5–10 min, 40% A; 10–15 min, 60% A; 15–18 min, 90% A. The absorbance of standard and sample solution was measured at 280 nm. Sample compounds were identified on the basis of retention times and absorption spectra of standard materials. Components were quantified by comparing their peak areas with those of standard curves.

#### Statistical analysis

All the assays were carried out in triplicate. Data were recorded as mean values and standard deviation (SD). The results were analyzed by Student’s *t* Test, using Microsoft^®^ Office Excel (Microsoft^®^, Redmond, Washington, USA), where values of *p* < 0.05 were considered as statistically significant.

## Results & Discussion

### Taxonomy

#### Russula senecis S. Imai

**Figure 1:**
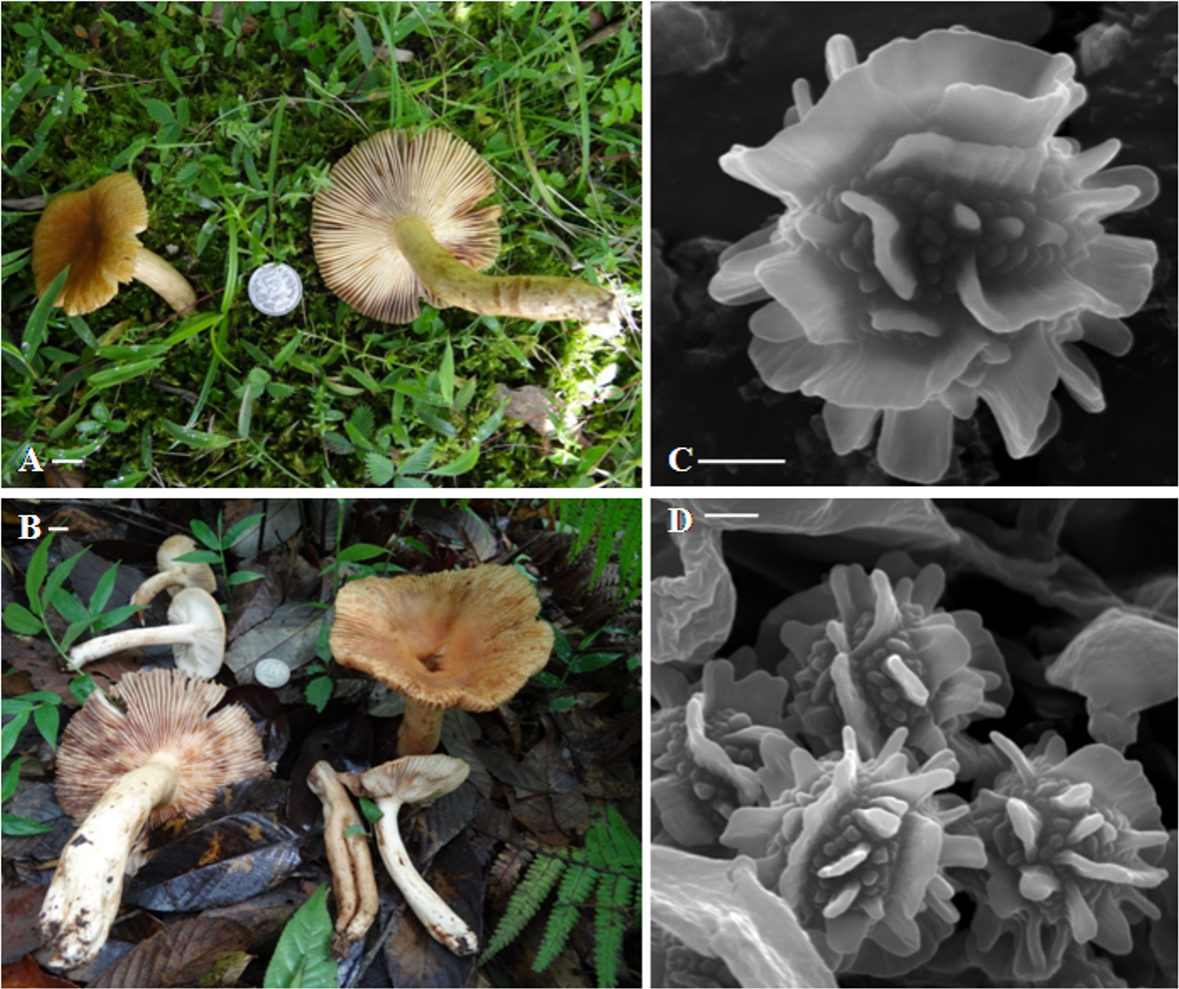
Fresh basidiomata and basidiospore ornamentation of *Russula senecis*. (A–B). Basidiomata. (C–D). SEM microphotograph of basidiospores. Bars (A–B): 10 mm; (C–D): 2 µm. Photographer for (A) and (B): Arun Kumar Dutta

**Figure 2:**
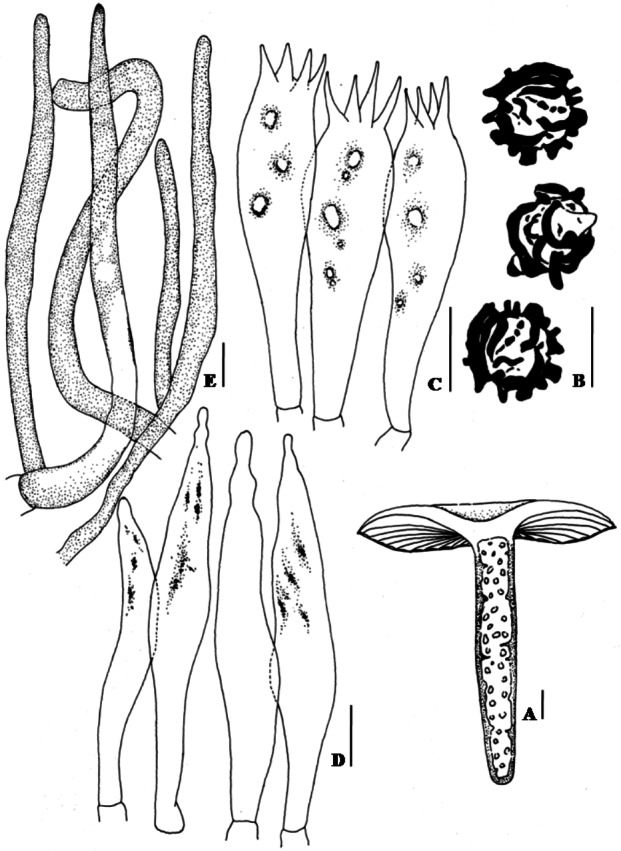
Hand drawing of macro- and microscopic characters of *Russula seneceis*. (A) Fresh basidiomata showing stipe context. (B) Basidiospores. (C) Basidium. (D) Hymenial cystidia. (E) Pileocystidia. Bars (A): 1 mm; (B–E): 10 µm.

**Pileus** 5.5–7(–13) cm broad, convex when young, becoming plano-convex to applanate at old, usually with broad central depression, glabrous, slightly viscid when wet, hygrophanous, bay, pale ochraceous buff to ochraceous-tawny towards centre, pallid to ochraceous buff towards margin, surface turns translucent rust to rusty-tawny with KOH; margin decurved, tuberculate striate; cuticle not easily separable from the context, cracking up into patches near margin; context up to 3.5 mm thick, creamy buff, unchanging color when exposed (Figs. 1A–1B). **Lamellae** 4.5–6 mm broad, adnexed, regular, bifurcate near the attachment of stipe, rarely one tiered, creamy buff, entire, even, edge discolorous, with fine brown to sienna buff margin. **Stipe** 5.5–7.5(–14) × 1.1–1.3(–2.4) cm towards top × 1.2–2.5 cm towards base, tapered towards the base, central to slightly eccentric, fleshy, slightly curved, cylindrical, becoming compressed, multi chambered at maturity; surface smooth, moist, slight shiny, creamy buff to dull yellow, often with fine dark brown warts, becoming clay buff on bruising, turns rusty-tawny to bay with KOH (Fig. 2A). **Odor** strong. **Taste** very acrid. **Spore print** creamy white.

**Figure 3:**
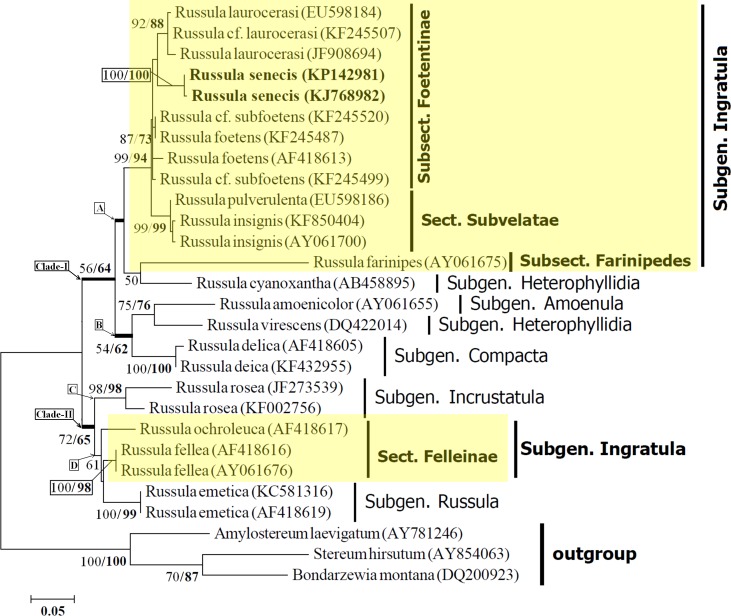
Maximum likelihood tree with the highest log likelihood (−2215.8014) generated using K2 + G model. The percentage of trees in which the associated taxa clustered together is shown next to the branches. The tree is drawn to scale, with branch lengths measured in the number of substitutions per site. Values to the left of/are Neighbour Joining bootstrap (BS) support, and those to the right indicate the ML bootstrap support of that clade. BS values >50% are shown. *Russula senecis* is placed in bold to highlight its phylogenetic position in the tree.

**Basidiospores** (7.5–)8.2–8.6–8.9(–9.7) × 7.8–8.3–8.6 µm, Q= 0.95–1.04–1.18, globose to subglobose, ornamentation amyloid, up to 2.1–3.2 µm high, composed of large wings and isolated warts, never forming reticulum (Figs. 1C and 1D; 2B). **Basidium** 32–38 × 10–10.7 µm, clavate, 4–spored (Fig. 2C). **Hymenial cystidia** (61–)64–68(–82) × 8.6–9.7(–10.7) µm, lanceolate to fusoid or elongated fusoid, with mucronate to moniliform apex, thin-walled, mostly with heteromorphous contents (Fig. 2D). **Lamellar trama** ca. 143–150 µm broad towards middle, 96 µm broad towards edge, mainly composed of sphaerocytes. **Subhymenium** pseudoparenchymatous. **Pileipellis** orthochromatic in cresyl blue, sharply delimited from underlying sphaerocytes of the context, distinctly divided into a dense, gelatinized, ca. 143–157(–161) µm deep subpellis composed of horizontally oriented hyphae, 3.2–3.6(–4.3) µm wide, mostly scattered with oleiferous fragments, (5.7–)6.4–7.2(–8.6) µm wide, and a less gelatinized, 36–72(–89) µm deep suprapellis of erect or repent hyphal ends. Incrustations absent. Pileocystidia up to 4.3–7.2 µm broad, mostly lanceolate, apex cylindrical to often with a minute rounded capitulum, thin-walled, recognizable by their distinct heteromorphous contents (Fig. 2E). Underlying sphaerocytes globose to sub-globose, ca. 12.5–13.9(–14.3) × 13.6–14.3 µm, hyaline. **Stipitipellis** up to 107–143 µm thick, composed of 3.6–3.9 µm broad hyphae, frequently with interspersed oleiferous hyphae, measuring 5.7–8.9 µm broad. **Caulocystidia** absent. **Stipe trama** composed of nested subglobose sphaerocytes, measuring 21–36(–44) µm diam.

Habit and habitat: common, ectomycorrhizal with *Shorea robusta* C.F.Gaertn. and *Castanopsis* sp.

Specimen examined: INDIA: West Bengal, Burdwan district, Malandighi, 11 July 2008, Prakash Pradhan, CUH AM103; Burdwan district, Malandighi, 25 August 2008, Prakash Pradhan, CUH AM104; Bankura district, Bishnupur, 10 August 2009, Prakash Pradhan, CUH AM105; Bankura district, Manjhulia, 15 July 2010, Prakash Pradhan, CUH AM106; Birbhum district, Gonpur, 08 July 2011, Arun Kumar Dutta and Prakash Pradhan, CUH AM107; East Midnapur district, Ramnagar, Kasaphaltalya, 24 July 2011, Arun Kumar Dutta and Prakash Pradhan, CUH AM108; Darjeeling district, Jawbari, 28 June 2012, Prakash Pradhan, CUH AM102; Darjeeling district, 7th mile Jungle, near Gurdum, 1 July 2012, Prakash Pradhan, CUH AM081.

**Notes:** *Russula senecis* was originally described as being from Japan (Imai, 1938), and reported to frequently grow in association with *Vateria indica* plants among the dipterocarp forests of Western Ghats (Natarajan et al., 2005), and in mixed forests under *Lithocarpus* and *Castenopsis* plant from Sikkim Himalaya, India (Das, 2009; Das, Van de Putte & Buyck, 2010). This well-known, widely distributed species can be easily recognized by the combination of an ochraceous-tawny pileus which turns rust to rusty-tawny with KOH, ochraceous buff tuberculate striate margin; creamy buff lamellae which often bifurcate near the attachment of stipe, discolorous lamellae with fine brown to sienna buff edges; creamy buff to dull yellow coloured, multi chambered stipe; acrid taste; strong odor; cream spore print; globose to sub-globose basidiospores (7.5–9.7 × 7.8–8.6 µm) with large wings and isolated warts, often with ridges (up to 2.1–3.2 µm high), but never form reticulum, absence of amyloid suprahilar spot; lanceolate to fusoid or elongated fusoid hymenial cystidia with mostly mucronate to moniliform apex; and lanceolate pileocystidia. The presence of these morphological features categorize *Russula senecis* within the subgen. *Ingratula* Romagn., series *Ingratae* (Quél.) Maire and subsect. *Foerentinae* (Melzer & Zvára) Singer (Sarnari, 1998).

Being a member of series *Ingratae* (of subgenus *Ingratula*), *R*. *senecis* closely resembles *Russula laurocerasi* and *Russula foetens*. However, *R*. *laurocerasi* differs from the present species by a light yellow to brilliant yellow or orange yellow coloured pileus with viscid to sticky surface, yellowish white lamellae, presence of lamellulae, pale yellow coloured spore-print, and up to 5 µm broad pileocystidia; and *R*. *foetens* differs by having characters like brilliant to dark or deep orange yellow or soft yellowish brown pileus, yellowish white coloured lamellae with lamellulae of two series, a stipe with veined surface, pale yellow spore-print, partially amyloid and mostly conic to acute tipped isolated warts basidiospores, and fusoid shaped hymenial and pileocystidia. A recently described species from India, *Russula dubdiana* K. Das, Atri & Buyck, differs from *R*. *senecis* by having a white coloured lamellae which turns sienna after bruising, white stipe when young, becoming faintly greying in places at maturity or hazel which turns fulvous to cinnamon towards base on bruising, smaller (5.2–7 × 4.2–5.5 µm) broadly ellipsoid to ellipsoid basidiospores with mostly of cylindrical warts and very few ridges and fertile lamellae edge (Das, Atri & Buyck, 2013).

### Molecular phylogeny

Phylogenetic analyses were performed on an ITS dataset of 28 sequences, of which 25 sequences were of the *Russula* species and the remaining three (*S*. *hirsutum*, *A*. *laevigatum*, and *B*. *Montana*) were used as an outgroup for rooting purposes. Sequencing products of the collected samples from different places in subsequent years ranged from 578 to 632 nucleotides. All sequences were aligned and the ends trimmed to create a dataset of 560 nucleotides that included 336 positions in the final dataset.

The resulting phylogram with the highest log likelihood value (–2215.8014) is represented in the present manuscript. The phylogram obtained using the Neighbor-Joining method displayed same topology with the phylogram obtained using ML analyses. Data obtained from the ML analyses and NJ analyses (Bootstrap percentage) has been indicated in Fig. 3.

Twenty five sequences of in-group *Russula* species distributed over five subgenus (Sarnari, 1998) resulted two distinct clades (I and II) with moderate bootstrap support (BS). Morphologically, all members of Clade-I are being well characterized by having basidiospores without an amyloid spot, whereas members under clade-II possess basidiospores with distinct amyloid spot. Clade-I is further subdivided into two subclades (viz. subclade-A and subclade-B) with 56% BS (NJ) and 64% BS (ML) respectively.

Within subclade-A, *Russula senecis* clusters with the members of the subsect. *Foetentinae* (viz. *R*. *laurocerasi*, *R*. cf. *laurocerasi*, *R*. *foetens* and *R*. cf. *subfoetens*) with high bootstrap support (99% BS and 94% BS) and is clearly separate from that of the sect. *Subvelatae*. Distinct differentiation of the subsect. *Foetentinae* from that of sect. *Subvelatae* based on the molecular data (ITS sequence) is also supported by the morphological characters such as the lack of reddening reaction with KOH and the absence of an arachnoid veil (Sarnari, 1998). *R*. *farinipedes* of the subsect. *Farinipedes* (subgen. *Ingratula*) clusters with that of *R*. *cyanoxantha* (subsect. *Cyanoxanthinae*; subgen. *Heterophyllidia*) with relatively low bootstrap support (50% BS in NJ analysis). Morphologically, both the species show white coloured spore-print, whereas species belonging to sect. *Subvelatae* and subsect. *Foetentinae* show a cream coloured spore-print. A similar result was also observed by Eberhardt (2002), where subsect. *Cyanoxanthinae* comes basal to the subsect. *Foetentinae* with bootstrap values >50%. In the present study, incorporation of species belonging to the section *Subvelate* results in a single clade with high bootstrap support values and subsect. *Cyanoxanthinae* (represented here by *R*. *cyanoxantha*) along with subsect. *Farinipedes* comes basal to the clade which contains members of subsect. *Foetentinae* and sect. *Subvelatae* (subgenus. *Ingratula*) with bootstrap values <50%.

*R*. *amoenicolor* and *R*. *virescens* cluster together and form subclade-B with 75% BS (NJ) and 76% BS (ML) respectively (morphologically, in both species number of lamellulae is rare) and clearly separates from that of *R*. *delica* (subgen. *Compacta*), generally known to posse’s abundant number of lamellulae (Sarnari, 1998), with 54% BS (NJ) and 62% BS (ML).

Clade-II consists of four species, distributed within three subgenus viz. *Incrustatula*; sect. *Felleinae* of subgen. *Ingratula*; and subgen. *Russula*. Member representing the subgenus *Incrustatula* (*R*. *rosea*) forms subclade-C and distinctly separates from that of subclade-D with moderate bootstrap supports (72% BS and 65% BS respectively). The separation of these two subclades within clade-II is also supported by morphological characters such as the presence (members belonging to subclade-D) or absence (species clusters within subclade-D) of pileocystidia.

Although sect. *Felleinae* is within the subgenus *Ingratula*, the present study reveals that sect. *Felleinae* (represented here by *R*. *fellea*) is more closely related to subgenus *Russula* than that of the remaining section *Subvelatae* and series *Ingratae* (subsect. *Foetentinae* and *Farinipedes*) of subgen. *Ingratula*. The discrete morphological difference of the members belonging to section *Felleinae*, is the presence of basidiospores with amyloid spot which is completely absent among the remaining sections of the subgen. *Ingratula* (Sarnari, 1998).

In accordance with the morphological features, phylogenetic analysis based on ITS1-5.8S-ITS2 sequence data revealed that, *R*. *senecis* clusters within the same clade (clade-A) together with that of *R*. *laurocerasi* and *R*. *foetens*, confirming its position within the same subsection *Foetentinae* under the series *Ingratae* of the subgen. *Ingratula*.

### Antioxidant activity

**Figure 4:**
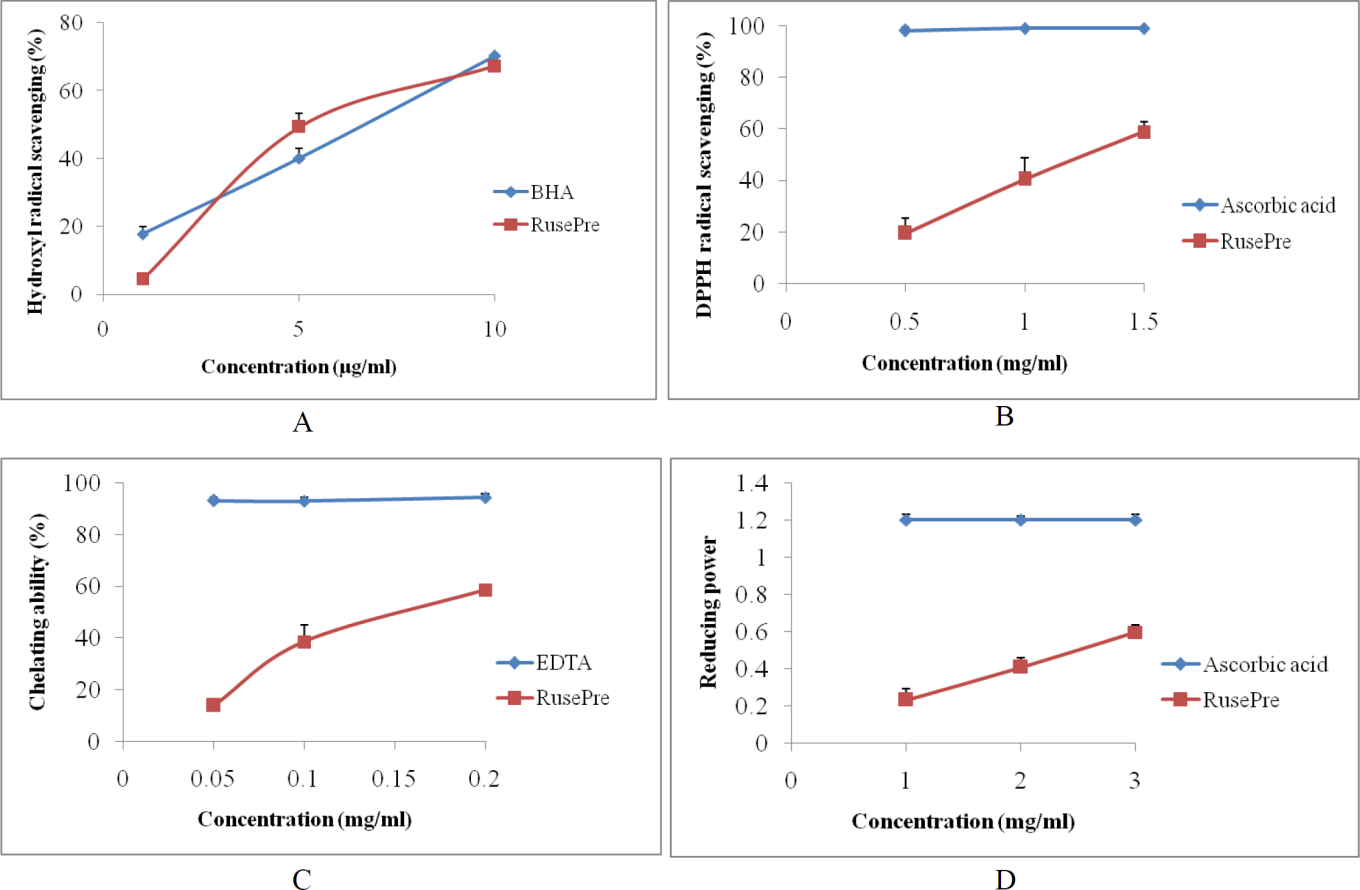
Antioxidant activity of phenol rich fraction from *Russula senecis* (RusePre). Results are presented as the mean ± SD of three separate experiments, each in triplicate. (A) Hydroxyl radical scavenging activity (B) DPPH radical scavenging activity (C) Chelating ability of ferrous ion (D) Reducing power.

In order to detect antioxidant activity, five biochemical assays were used: total antioxidant capacity (based on reduction of Mo(VI) to Mo(V) by antioxidant compound and formation of green phosphate/Mo(V) complex), inhibition effects on hydroxyl radicals (measures color intensity of MDA-TBA complex which decreases in presence of radical scavengers), scavenging effects on DPPH radicals (determines decrease in absorbance of DPPH solution accompanying with antioxidants), chelating ability of ferrous ions (deals with binding capacity of antioxidant with ferrous ions) and reducing power (decides electron donation ability of antioxidant which converts Fe^3+^/ferricyanide complex to Fe^2+^). The results are expressed graphically in Fig. 4. Total antioxidant capacity assay indicated that 1 mg of RusePre acted as the equivalent to 13.44 ± 0.67 µg of ascorbic acid. Moreover, RusePre extract proved to be more active as hydroxyl radical scavengers and iron chelators. The EC_50_ values were 5 ± 0.2 µg/ml and 158 ± 10 µg/ml for hydroxyl radical scavenging and chelating ability of ferrous ion respectively suggesting extremely high activity of the extract. In addition, it was an effective antioxidant as a DPPH radical scavenger, as evident by the low EC_50_ value (1.34 ± 0.07 mg/ml). Investigation also revealed that RusePre had a high reducing ability which increased in a dose dependent manner (EC_50_ value 2.495 ± 0.015 mg/ml). In the above four cases, differences between RusePre and the control were found to be statistically significant (*p* < 0.05) except for hydroxyl radical scavenging activity. Recently, antioxidant activities of phenol rich extracts of some wild edible mushrooms such as *Russula albonigra* (Krombh.) Fr. (RalPre) (Dasgupta et al., 2014) and *Amanita vaginata* (Bull.) Lam. (AvaPre) (Paloi & Acharya, 2013) have been reported. In comparison, the measured activities of RusePre were found to be higher than AvaPre but lower than RalPre.

### Antimicrobial activity

The antimicrobial effect of RusePre was tested against six species of pathogenic bacteria. Moderate inhibitory effect was found against *L. monocytogenes*, *B. subtilis*, *P. aeruginosa* and *S. aureus* and the inhibition zones were between 5 mm to 7 mm. However, RusePre was ineffective against *E. coli* and *S. typhimurium*, as the inhibition zones were <5 mm. It may be expected that the antimicrobial activity of fraction would be related to its phenolic compounds. Our finding was somewhat similar with the ethanolic fraction of *Russula delica*, as it was effective against *Bacillus cereus*, *L. monocytogenes* and *S. aureus*. On the other hand, *E. coli*, *P. aeruginosa* and *Salmonella enteritidis* were inhibited very weakly (Yaltirak et al., 2009).

### Chemical composition

The extractive yield of brown colored RusePre was 36 ± 2%. To investigate the chemical nature of RusePre, different parameters such as phenol, flavonoid, *β*-carotene, lycopene and ascorbic acid were tested. Results showed that phenol was the major naturally occurring antioxidant component and value was 14.142 ± 1.05 µg gallic acid equivalent/mg of extract. RusePre also contained a flavonoid as 4.427 ± 1.123 µg quercetin equivalent/mg of extract. Very negligible amounts of *β*-carotene and lycopene were found such as 0.633 ± 0.01 µg/mg and 0.59 ± 0.01 µg/mg of the extract respectively. Ascorbic acid was also present in small quantities, and the obtained value was 1.22 ± 0.17 µg/mg of dry extract. Puttaraju et al. (2006) have reported phenolic content of water and methanol extract of *Russula brevipes*, and the recorded values were 5.5 and 0.7 µg gallic acid equivalent/mg of sample. The total phenolic and flavonoid contents of methanolic extract of *R. delica* were 2.09 µg gallic acid equivalent/mg of extract and 0.16 µg quercetin equivalent/mg of extract (Gursoy et al., 2010). Thus, it can be assumed that our extraction procedure was appropriate to produce a fraction with adequate phytochemicals.

**Figure 5:**
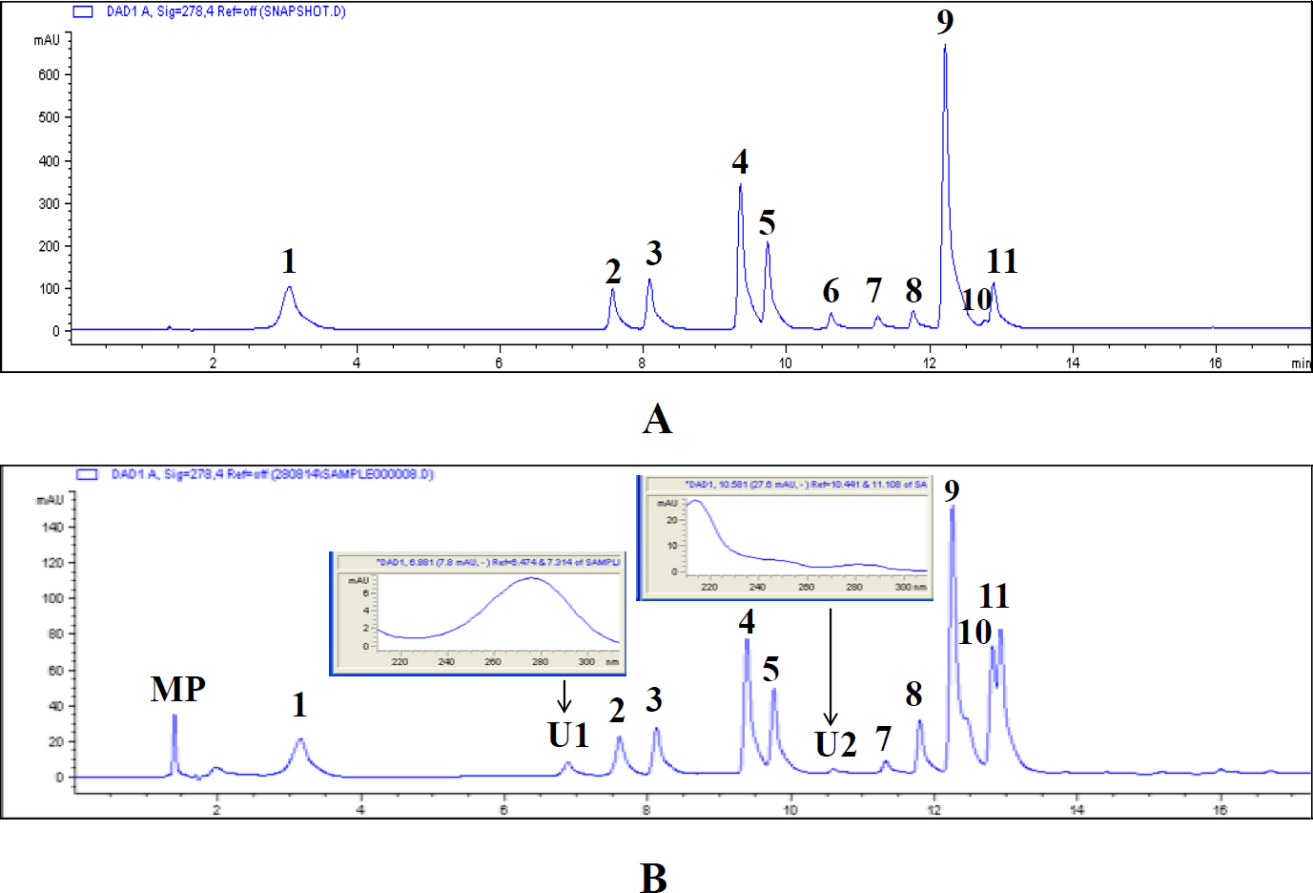
HPLC chromatogram of standards and phenol rich extract of *Russula senecis* (RusePre). (A) Standards each in 50 µg/ml concentration (peaks: 1, gallic acid; 2, chlorogenic acid; 3, vanillic acid; 4, *p*-coumaric acid; 5, ferulic acid; 6, myricetin; 7, salicylic acid; 8, quercetin; 9, cinnamic acid; 10, pyrogallol; 11, kaempferol) (B) Phenolic profile of RusePre with UV spectra of two unidentified peaks (inset) (MP, mobile phase; 1, gallic acid; U1, unidentified peak 1; 2, chlorogenic acid; 3, vanillic acid; 4, *p*-coumaric acid; 5, ferulic acid; U2, unidentified peak 2; 7, salicylic acid; 8, quercetin; 9, cinnamic acid; 10, pyrogallol; 11, kaempferol).

Furthermore, the molecular phenolic profile of RusePre was determined using HPLC-UV, an important tool for quantitative analysis (Sheikh et al., 2014; Liu et al., 2013). Figure 5A depicts a typical HPLC chromatogram of eleven phenolic compounds each at 0.05 mg/ml concentration, and Fig. 5B represents HPLC chromatogram of RusePre at 0.5 mg/ml concentration. The results showed a qualitative profile of RusePre which was composed of all standard phenolic compounds except myricetin and two unrecognized phenolic substances (*λ*max in inset). Quantitatively pyrogallol was present in the highest amount (Table 1). Overall, flavonols (166.01 µg/mg of dry extract) along with cinnamic acid and its derivatives (106.15 µg/mg of dry extract) might be the main contributors in phenolic profile rather than hydroxybenzoic acid derivatives (73.59 µg/mg of dry extract). Thus, it can be assumed that RusePre might be enriched with flavonols and hydroxycinnamic acid derivatives. The present finding is also supported by various similar studies. Puttaraju et al. (2006) reported tannic acid, protocatechuic acid, gallic acid, gentisic acid, vanillic acid, *p*-coumaric acid and syringic acid in the phenolic composition of water and methanol fraction from *Russula brevipes* Peck, whereas Ribeiro et al. (2006) informed the presence of *p*-hydroxybenzoic acid in *Russula cyanoxantha* (Schaeff.) Fr. Subsequently, gallic acid, caffeic acid and rutin from *Russula delica* Fr.; cinnamic acid from *Russula caerulea* Fr. and *Russula sardonia* Fr. had also been detected (Alves et al., 2013).

**Table 1:** Phenolic profile of phenol rich extract of *Russula senecis* (RusePre).

Peak no.	RT (min)	*λ*max (nm)	Area	Concentration (μ g/mg)	Compound
1	3.146	272	378.6	22.31	Gallic acid
U1	6.879	276	77.1	Not identified	Not identified
2	7.599	273	201.2	29.63	Chlorogenic acid
3	8.117	260, 295	247.5	25.72	Vanillic acid
4	9.376	310	572.5	23.43	*p*-coumaric acid
5	9.753	290, 325	401	26.55	Ferulic acid
U2	10.581	284	24.1	Not identified	Not identified
6	10.627	250, 373	Not identified	Not identified	Myricetin
7	11.314	303	57	25.56	Salicylic acid
8	11.792	255, 372	203.5	79.83	Quercetin
9	12.244	277	1322.6	26.54	Cinnamic acid
10	12.8	276	403.3	133.9	Pyrogallol
11	12.918	265, 365	723.5	86.18	Kaempferol

## Conclusion

The DNA barcoding and therapeutic value of *Russula senecis*, a wild mushroom exclusively consumed by ethnic people of West Bengal, was unexplored in the scientific world until this work. For the first time a complete ITS region of *R*. *senecis* has been sequenced and its taxonomic position within the subsection *Foetentinae* under the series *Ingratae* of the subgen. *Ingratula* has been supported with molecular phylogenetic analysis. To determine its medicinal properties, a heat stable phenol rich extract (RusePre) was prepared using water and ethanol as solvent system. Results clearly indicated that RusePre has antioxidant activity against various *in vitro* systems, even after the heat treatment. The fraction showed extreme potentiality in scavenging hydroxyl radical and chelating ability of ferrous ion than DPPH radical scavenging, reducing power and total antioxidant method. Furthermore, administration of RusePre inhibited several pathogenic bacteria such as *Listeria monocytogenes*, *Bacillus subtilis*, *Pseudomonas aeruginosa* and *Staphylococcus aureus*. The pronounced activity was possibly due to its high phenol and flavonoid content in addition with carotenoids and ascorbic acid which were presented in minor amounts. Molecular phenolic profiling of RusePre by HPLC-UV indicated probable existence of at least 13 phenolics of which 10 were identified such as pyrogallol, flavonols (Kaempferol, quercetin), benzoic acid derivative (vanillic acid> salicylic acid> gallic acid), cinnamic acid and its derivatives (chlorogenic acid> ferulic acid, cinnamic acid> *p*-coumaric acid). Thus, the studied mushroom may have great potential for food and nutraceutical industries as a source of bioactive molecules such as phenolic components for dietary supplements and functional food.

## Additional Information and Declarations

### DNA Deposition

The following information was supplied regarding the deposition of DNA sequences: GenBank: KP142981, KJ768982.
